# Full-Sibs in Cohorts of Newly Settled Coral Reef Fishes

**DOI:** 10.1371/journal.pone.0044953

**Published:** 2012-09-13

**Authors:** Giacomo Bernardi, Ricardo Beldade, Sally J. Holbrook, Russell J. Schmitt

**Affiliations:** 1 Department of Ecology and Evolutionary Biology, University of California Santa Cruz, Santa Cruz, California, United States of America; 2 Universidade de Lisboa, Faculdade de Ciências, Centro de Oceanografia, Campo Grande, Lisboa, Portugal; 3 Department of Ecology, Evolution, and Marine Biology and The Marine Science Institute, University of California Santa Barbara, Santa Barbara, California, United States of America; Leibniz Center for Tropical Marine Ecology, Germany

## Abstract

Reef fishes exhibit a bipartite life cycle where a benthic adult stage is preceded by a pelagic dispersal phase during which larvae are presumed to be mixed and transported by oceanic currents. Genetic analyses based on twelve microsatellite loci of 181 three-spot dascyllus (*Dascyllus trimaculatus*) that settled concurrently on a small reef in French Polynesia revealed 11 groups of siblings (1 full sibs and 10 half-sibs). This is the first evidence that fish siblings can journey together throughout their entire planktonic dispersal phase (nearly a month long for three-spot dascyllus). Our findings have critical implications for the dynamics and genetic structure of fish populations, as well as for the design of marine protected areas and management of fisheries.

## Introduction

Most reef fishes exhibit a bipartite life cycle with a sedentary benthic adult phase and a pelagic larval phase. The pelagic phase, with a duration that can vary from several days to a few months, is the dispersive phase when larvae are presumed to be mixed and transported by oceanic currents [1]. At the end of the pelagic stage, larvae settle on the reef. This view of the life cycle – with mixing and transport during the pelagic phase - has fundamental ramifications for how we envision the dynamics and genetic structure of fish populations, together with all the practical implications that ensue, such as the design of marine protected areas [2] and the management of fisheries resources [3].

The analysis of genetic relatedness of cohorts of settling recruits can yield important spatial, temporal and mechanistic insight into patterns of larval dispersal in marine organisms [4]–[7]. One important realization that may come from such studies regards the number of adults that effectively contribute to recruitment [8]–[10]. According to the ‘sweepstakes effect’ only a small proportion of the available gene pool successfully contributes to the replenishment of the population [8]. Under this hypothesis, one should thus expect to find less genetic diversity within cohorts than among them [9]. Indeed, significant changes in allele frequencies of larvae or recruits have been documented from one sampling time to the next [4], [6], [11]–[14].

Genetic fingerprinting, through highly variable molecular markers used in parentage analysis assessments, has enabled the tracking of marine fish larvae [10], [15]. Compared to other marking methods of tracking marine fish larvae such as otolith marking [16], genetic methods offer the ability to identify and track individual fish. The combination of molecular methods and water circulation patterns has been used to highlight the likely pathways taken by individual larvae [15], [17], [18].

Three-spot dascyllus, Dascyllus trimaculatus, display a typical life cycle for a coral reef damselfish, where males defend a territory and attract females that spawn on the substrate. Eggs are guarded for two to three days until they hatch [19]–[21]. After approximately 22–26 days in the water column [22], larvae settle at night on sea anemones [23]–[27] where they remain until the sub-adult stage. Adults do not occupy anemones but shelter in nearby reef crevices. There are two 3- to 5-day-long pulses of settlement around the quarter moons of each lunar month, with relatively little settlement in between (Figure S1) [25]. The predictability of settlement in D. trimaculatus, and the ease of manipulation for the settlement substrate (anemones) make this system conducive to studies of recruitment dynamics.

For this study, we established an array of 16 sea anemones on the north shore of Moorea, French Polynesia, and collected newly settled D. trimaculatus every day during two recruitment pulses in November and December 2004. We used relatedness analysis based on highly variable microsatellite markers to identify genetically related recruits in space (anemones) and time (different recruitment pulses or days), combined with mitochondrial DNA sequences to identify maternal ancestry of the recruits. We found that related individuals mostly recruited to the same or nearby anemones on the same night, indicating that they remained together during their entire pelagic phase.

## Results

### Relatedness Analysis Based on Microsatellites

The analysis of microsatellites in adult populations of Dascyllus trimaculatus has shown that the markers that we used in this study are in Hardy-Weinberg equilibrium and do not show signs of linkage disequilibrium [28], [29]. Here we used the twelve microsatellites to analyze relatedness in 181 newly settled individuals. While the markers showed the expected levels of genetic diversity (average number of alleles was 15.2, Table 1), our prediction that some individuals would be related is consistent with the observation of lower levels of observed heterozygosity compared to the expected heterozygosity for all microsatellite loci, with a mean observed heterozygosity of 0.63 and a mean expected heterozygosity of 0.68 (Table 1).

**Table 1:** *Dascyllus trimaculatus* microsatellite characteristics based on recruits (N = 181, this study).

Locus	N_a_	H_o_	H_e_
DTR_A7	5	0.50	0.52
DTR_A101	31	0.73	0.79
DTR_A103	22	0.77	0.79
DTR_A105	13	0.78	0.80
DTR_A111	7	0.73	0.78
DTR_A114	17	0.67	0.83
DTR_A115	16	0.80	0.80
DTR_A120	24	0.77	0.83
DTR_B103	30	0.81	0.92
DTR_B105	9	0.64	0.63
DTR_B109	3	0.18	0.25
DTR_B113	6	0.19	0.19

Columns correspond to: microsatellite name (Locus), number of alleles (N_a_), observed (H_o_) and expected (H_e_) heterozygosities.

We assessed globally whether individuals in the sample were genetically more related than expected by testing the null hypothesis of no relatedness against the distribution of the moment of pairwise relatedness coefficients in the observed population. We found that individuals we collected were more genetically related than expected by chance alone. Indeed, a t-test on the mean relatedness observed and those obtained through a permutation procedure revealed statistically significant differences between them (observed mean rxy = 0.010, resampled mean rxy = 0.0071, t = 1173, P<0.001) (Figure S1).

**Figure 1:**
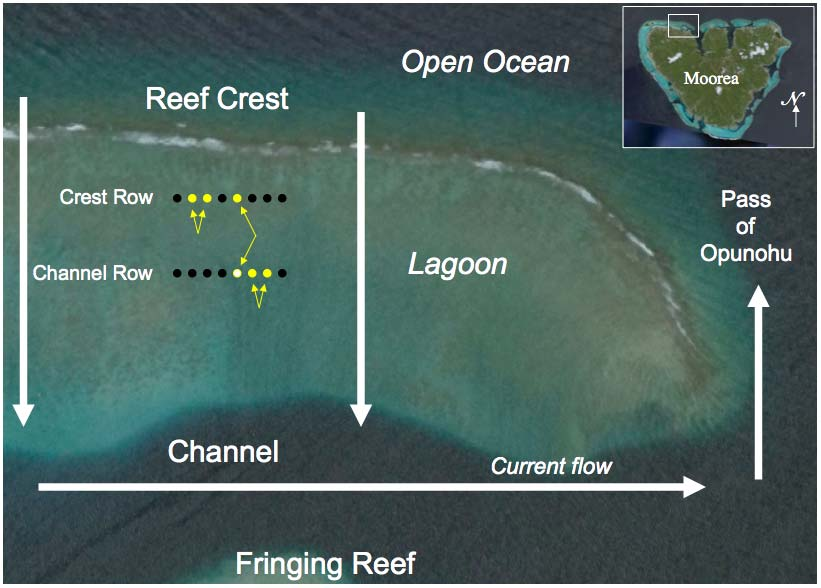
Research site located on the northwestern shore of the island of Moorea, French Polynesia (inset). General water circulation is from the open ocean, over the barrier reef, towards the channel, and then out of the lagoon through the pass (white arrows). We placed 2 rows of 8 anemones (black dots), a Crest Row and a Channel Row. One pair of full sibling fishes was found on Channel Row anemone number 5 (white dot). Two pairs of half siblings were found on adjacent anemones, Channel Row anemones 6 and 7, Crest Row anemones 2 and 3 (yellow dots and yellow arrows). One threesome of half sibs included two individuals in one anemone (channel 5) and one individual on the Crest Row (anemone 5), indicated by yellow and white dots and yellow arrows.

Pairwise relatedness analysis revealed a large number of individuals that were potentially related. Out of 16,290 pairwise comparisons, 14 corresponded to R indexes [30] above 0.5 (comprised between 0.50 and 0.60). Those 14 pairwise comparisons corresponded to 8 pairs of individuals and 3 threesomes, with assignable and unique genetic and settlement patterns (Table 2). The members of each of the 11 groups settled from the plankton onto the reef on the same night. One pair settled on the same anemone (Channel 5). Two individuals of one threesome also settled on the same anemone (Channel 5), the third individual of that threesome having settled on the Crest 5 anemone, which is just upstream from the Channel 5 anemone (Figure 1). Individuals from two pairs settled on adjacent anemones (Figure 1). Individuals from four other pairs settled on anemones that were on the same row, and two additional individuals that settled elsewhere were included in two of those groups, thus forming threesomes (Table 2). The remaining two pairs had individuals that settled on Crest and Channel Rows (Table 2). Overall, we obtained more related individuals on the inshore Channel Row than the offshore Crest Row (Figure 1, Table 2), however this pattern did not differ significantly from that of overall settlement to the two rows of anemones (Chi square, P>0.1), thus settlement did not preferentially occur on one row or the other.

**Table 2:** Dascyllus trimaculatus siblings.

		Locus	A7	A101	A103	A105	A111	A114	A115	A120	B103	B105	B109	B113
R	SampleName													
	**Full sib**													
0.6	IN05_01		10,10	11,24	15,17	21,23	25,27	43,44	09,26	10,10	10,18	04,04	08,08	03,03
	IN05_09		10,10	11,24	15,17	20,20	25,26	27,44	09,26	10,17	10,18	04,04	08,08	03,11
	**Half sib - same anemone**													
0.53	IN05_03		07,10	09,09	15,15	23,29	24,25	28,39	09,15	17,17	11,14	04,06	08,08	03,03
	IN05_04		10,10	09,09	15,15	23,23	27,27	28,40	13,15	17,17	11,14	04,06	08,08	03,11
	OUT05_01		07,10	09,09	15,15	20,20	25,28	49,49	09,15	17,17	13,16	04,06	08,08	03,03
	**Half-sib - adjacent anemone**													
0.53	IN06_02		10,11	09,09	15,31	22,25	27,27	28,43	09,25	14,17	19,28	04,04	08,08	03,03
	IN07_04		11,11	09,24	15,15	22,30	27,27	40,43	09,25	10,17	10,28	04,06	08,08	03,03
														
0.5	OUT02_02		10,10	09,21	15,15	22,22	27,27	27,44	15,15	10,41	21,23	05,06	09,09	03,12
	OUT03_06		10,10	09,18	15,15	22,22	25,25	27,42	15,26	10,16	10,21	04,06	09,09	03,04
	**Half-sib - same row**													
0.5	IN04_04		10,10	11,24	15,26	22,25	27,27	43,43	15,26	15,24	11,12	04,06	08,08	03,09
	IN06_07		10,10	11,11	15,15	22,23	23,27	43,47	09,26	15,24	11,11	04,04	08,08	03,03
														
0.53	IN04_15		10,11	11,11	15,15	23,25	28,28	27,42	15,15	10,17	13,13	04,04	08,08	03,03
	IN07_10		10,11	09,11	15,18	22,23	28,28	27,28	09,09	10,10	13,14	04,04	08,08	03,03
														
0.52	IN03_03		10,10	12,12	15,21	20,29	26,28	27,43	21,25	17,17	12,23	04,04	08,08	03,03
	IN07_01		10,10	09,12	13,19	11,29	26,28	27,27	09,21	17,17	10,13	04,06	08,08	03,03
	OUT02_05		10,10	09,11	15,25	20,20	25,28	42,45	21,25	17,17	06,12	04,04	08,08	03,03
														
0.54	IN01_06		10,10	09,09	15,23	29,29	26,28	27,27	15,25	24,25	17,28	06,07	08,09	03,03
	IN04_08		10,10	09,09	15,15	22,22	26,28	27,27	09,26	15,17	11,25	04,06	08,08	03,03
	OUT05_03		10,10	09,09	15,15	11,22	28,28	27,27	15,25	24,24	16,22	04,06	08,08	03,03
	**Half-sib different rows**													
0.55	IN06_02		10,11	09,09	15,31	22,25	27,27	28,43	09,25	14,17	19,28	04,04	08,08	03,03
	OUT03_04		10,10	09,11	24,31	22,25	27,27	43,45	25,25	17,27	11,12	04,04	08,08	03,03
0.51	IN02_12		10,11	09,09	15,21	29,29	25,28	27,27	15,24	17,25	11,12	04,05	08,08	03,04
	OUT05_04		10,11	09,09	15,16	22,29	28,28	27,27	25,25	17,17	12,12	04,04	08,08	03,03
0.51	IN05_07		10,10	10,11	15,15	22,29	27,27	44,44	25,25	17,24	11,16	04,04	08,08	03,03
	OUT03_04		10,10	09,11	24,31	22,25	27,27	43,45	25,25	17,27	11,12	04,04	08,08	03,03

Labels indicate the location of sampling (IN Channel Row, OUT Crest Row) the anemone number and the individual number (e.g. IN05_01, Channel anemone 5, individual 1). Pairs of numbers indicate the repeat numbers of microsatellite alleles (e.g. 10,10, is a homozygote with 10 repeats for both alleles). The left column indicates the relatedness index (R), the first row indicates the name of the microsatellite loci.

#### Mitochondrial DNA analysis

Previous work on Dascyllus trimaculatus revealed that in French Polynesia, adult individuals show high levels of control region genetic diversity, where out of 61 sequenced individuals, only 5 shared a mitochondrial haplotype [31]. In this study, haplotype diversity was also very high (0.988), and the typing of 329 Moorea individuals (181 recruits and 148 adults) resulted in 159 haplotypes (not shown). Out of the 181 sequences obtained from the recruits, we found that 7 pairs of individuals and one threesome that recruited the same night on the same anemone also shared the same haplotype (8 different haplotypes, a unique haplotype for each pair and a unique haplotype for the threesome). Of the 11 groups of related individuals identified by microsatellite analysis and described above, one pair (Pair 1, Table 2) was among those that also shared the same mitochondrial haplotype. Individuals of Pair 1 shared a haplotype that was not found in any other individual. The probability for those two individuals to share this haplotype was therefore (2/181)^2^, a probability of P = 1.22 10^−4^ The other pairs of individuals did not share a mitochondrial haplotype nor did they share a haplotype with individuals that settled with them on the same anemone or during the same night (not shown).

## Discussion

In our study, we found that at least 25 out of 181 recruits (14%) are related, yet these results identify the minimum number of related individuals in the sample and do not mean that the remaining 86% are unrelated. It is likely, in fact, that additional individuals are also related, but our data do not have the power to determine how many such individuals are present.

The power of our study comes from a multi-pronged approach. Indeed, ecological, microsatellite, and mitochondrial data respectively yielded insight on the timing of recruitment, relatedness, and the maternal lineage of individual three-spot dascyllus. Our results revealed that individual three-spot dascyllus that were genetically related recruited to the reef together following their nearly month-long planktonic larval duration. One pair (Pair 1) of related individuals that recruited on the same night in the same anemone also shared the same mitochondrial haplotype. In addition, this pair displayed the highest relatedness value of the entire dataset (0.6, Table 2). These individuals are therefore likely to be full sibs (from the same clutch), as the sharing of mitochondrial haplotypes is highly unlikely to occur by chance alone. Indeed, we estimated the likelihood for individuals with identical haplotypes to settle on the same anemone at the same time was less than 10^−6^. The second category of individuals, which did not share the same mitochondrial haplotypes, most likely corresponded to individuals that are genetically very close. The most parsimonious explanation for these individuals is that they are half-sibs. The most likely ecological scenario is for those individuals to share the same father and have different mothers. Indeed, male three-spot dascyllus are often observed courting several females during the same spawning sessions, and it is likely that several females deposit eggs in the same nest on the same day and for the eggs to later hatch at the same time (authors obs.). The half-sibs we identified likely hatched from the same nest and journeyed together throughout their planktonic period.

Finding that siblings recruit together is consistent with the idea that dispersing fish larvae from the same clutch possess sensory and behavioral mechanisms that enable them to remain in very close proximity of each other throughout their planktonic dispersal phase [32]. This was argued to occur in a closely related damselfish species, Dascyllus aruanus [7]. In that study, relatedness of 265 individuals collected in lagoons of Moorea was assessed based on 10 microsatellite markers. High levels of relatedness in 35 pairs of individuals that also had similar body sizes were suggestive of a genetic relationship. However, as these were all individuals that had been on the reef for some time at the time of their collection, it was not known if they recruited together on the same day. In addition, information on maternal ancestry was not available [7].

Mechanisms that result in dispersing propagules remaining together can create potentially important homogeneity in larval recruitment. For example, heightened population genetic structure has repercussions for estimates of dispersal based on genetic models that assume that propagules are well mixed, which has fundamental implications for the design of Marine Protected Areas [2]. From an ecological perspective, reduction in the degree of larval mixing implies that spatial heterogeneity in fecundity can translate into heterogeneity in settlement due to reduced spatial averaging during the dispersal phase. The movement of water masses in coastal systems can show high spatial and temporal autocorrelation, which decreases the total number of independent larval trajectories [33], [34].

The use of relatedness analysis to study recruitment patterns is a rapidly developing field that also comes with some inherent complexities [35], [36]. For example, Queller and Goodnight’s R index that we used in our study is only one of several ways to assess relatedness. Here, we decided to use a very conservative approach by not considering a large amount of data that pointed towards genetic relationship of recruiting individuals but instead restricted our analysis to only those pairs with the highest R indices. It is possible that a larger number of individuals in our sample were genetically related, a result that is also consistent with the observed low heterozygosities shown in Table 1. Additional samples and more powerful genetic markers, such as large numbers of Single Nucleotide Polymorphisms (SNPs), would allow more power to determine the true fraction of related individuals [37]. Thus far, studies of relatedness in reef fish have mostly focused on self-recruitment in anemonefishes, whereby some individuals return to the reef where they were born at the end of the planktonic period. Anemonefishes have a shorter pelagic larval duration and exhibit, in general, smaller population sizes than D. trimaculatus. Our findings suggest that even in species with longer pelagic larval durations and larger population sizes, relatedness studies give an opportunity to explore additional aspects of recruitment dynamics.

The paradigm that cohorts of larval fishes are well-mixed and highly dispersed was first challenged by findings of self-recruitment at the 1 to 10 kilometer scale [38], [39], and later confirmed by additional genetic information of anemonefishes [15]. This study adds further evidence that larval fishes are not always a genetically well-mixed pool. Regardless of the distance fish larvae move from their natal reef, individuals of at least some species can remain together from birth to settlement despite relatively long planktonic durations.

## Materials and Methods

### Field Research

All necessary permits were obtained for the described field studies from the French Polynesia Ministry of Research to the authors, and in accordance with University of California Santa Barbara’s Institutional Animal Care and Use (IACUC) Protocol # 639 and University of California Santa Cruz IACUC Protocol # Berng1101. The research presented in this manuscript was approved by our Institutional IACUC Committee.

### Assay Design

This study was performed on the northwestern shore of Moorea, French Polynesia (Figure 1). In this area, waves break over the crest of the barrier reef creating a shoreward current inside the lagoon that reaches a channel that is parallel to the barrier reef [40]. Water then moves eastward alongshore and then northward to exit the nearby pass of Opunohu (Figure 1). Thus the likely trajectory of larvae in the ocean just offshore of the barrier reef is first to enter the lagoon over the crest, and then follow the current shoreward in the lagoon. During this passage, larvae settle on anemones at night. Larvae that do not settle are transported outside the lagoon by the outward currents in the pass (Figure 1).

The location for our assay was chosen for its initial lack of anemones and adult *Dascyllus trimaculatus*, to avoid any confounding effect of recruitment on any nearby unsurveyed anemones. Sixteen anemones collected in November 2000 on the north and western shores of Moorea were placed at our study site in two parallel rows of eight: one row just inshore from and parallel to the barrier reef (Crest Row), and a second row 100 meters inshore from the Crest Row (Channel Row, Figure 1). Anemones were spaced 10 meters apart to avoid movement of young recruits between anemones [41]. Settlement of three-spot dascyllus occurred regularly on the anemones in the period prior to our assays. Before the beginning of each of our assays, all previously-settled damselfishes were removed from the anemones. Then, every morning during a four-week-long recruitment period, we collected all newly settled fish from the previous night, starting before the recruitment bout (Figure S2). Overall 181 newly settled recruits (Channel Row: 122; Crest Row: 59) were used in our genetic analyses, encompassing two discrete recruitment pulses during the months of November and December 2004 (Figure S2).

### Molecular Analysis

Fishes were genotyped using highly variable molecular markers: the mitochondrial DNA control region (protocol in [31]) was used to identify maternal ancestry. Number of haplotypes and haplotype diversity were calculated using the software package DNAsp [42]. Twelve microsatellites [28], [29] were used for relatedness analysis. Data were tested for Hardy-Weinberg equilibrium, linkage disequilibrium, and the presence of null alleles, large-allele dropout or stuttering using Arlequin 3.5 [43] and Micro-checker [44]. Expected and observed heterozygosities, number of alleles per locus and allelic richness were calculated with Arlequin 3.5 [43]. Only those individuals where all loci were successfully amplified and scored were used in the analysis.

### Relatedness Analysis

We assessed the degree of relatedness between individuals using the relatedness R index [30], as implemented in Identix [45]. R is based on the number of shared alleles between pairs of individuals standardized according to the individual’s state (homozygous or heterozygous) and the allelic frequency in the sampled population. This index varies between 0 and 1, with 0.5 corresponding to full-siblings. In order to determine globally whether individuals in the sample were genetically more related than expected, given that their parents had mated randomly, we tested the null hypothesis of no relatedness by comparing the distribution of the moment of pairwise relatedness coefficients in the observed population with its null expectation. This null distribution was obtained by a conventional Monte Carlo resampling procedure, which randomly selected 10,000 genotypes without replacement and then recalculating the statistic. H_o_ could be rejected with a significance level of 5%, given that the observed value of the observed statistic was above the 95% level of the resampled statistics. The arithmetic mean of the pairwise relatedness coefficients from the observed sample matrix was compared directly to the proportion of resampled means to determine whether individuals within this sample are genetically more related than expected under the null hypothesis (Figure S1). The mitochondrial control region was also sequenced for all individuals and given that mtDNA is maternally inherited, provided information of maternal lineage. To analyze spatial distribution of full-siblings, the location and recruitment date of each full-sibling were mapped.

## Supporting Information

Figure S1 **Test of the null hypothesis of no relatedness was done by comparing the distribution of the moment of pairwise relatedness coefficients in the observed population with its null expectation.** Null distribution was obtained by a conventional Monte Carlo resampling procedure, which randomly selected 10,000 genotypes without replacement and then recalculating the statistic. We found that individuals we collected were more genetically related than expected by chance alone. A t-test on the mean relatedness observed and those obtained through a permutation procedure revealed statistically significant differences between them. Observed mean rxy = 0.010 is indicated by an arrow, resampled mean rxy = 0.0071, t = 1173, P<0.001.
(TIFF)

Figure S2 **Recruitment of** ***Dascyllus trimaculatus.*** Plot of number of recruits collected each day of a recruitment cycle in Moorea, French Polynesia. Dates of the month of November and December are on the x axis, number of collected recruits are on the y axis. Phases of the moon are indicated showing a peak recruitment at the quarter moon and a low recruitment at full and new moons. Channel Row and Crest Row recruitments are shown separately in blue and red, respectively.
(TIFF)
