# Supplementary figures and images for: Full-Sibs in Cohorts of Newly Settled Coral Reef Fishes
Source: PLoS One. 2012 Sep 13;7(9):e44953. doi: 10.1371/journal.pone.0044953 (PMC3441696; doi:10.1371/journal.pone.0044953)

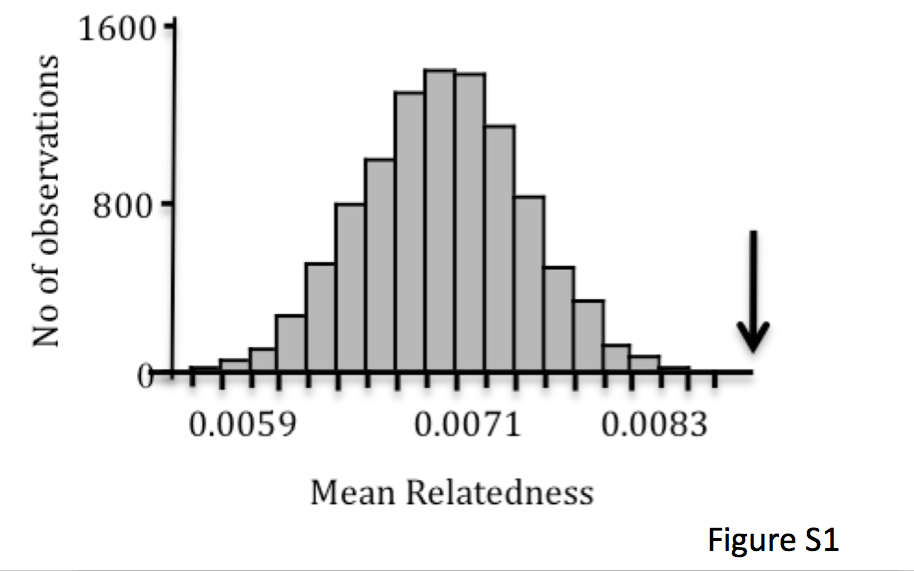

Supplement: Figure S1 — Test of the null hypothesis of no relatedness was done by comparing the distribution of the moment of pairwise relatedness coefficients in the observed population with its null expectation. Null distribution was obtained by a conventional Monte Carlo resampling procedure, which randomly selected 10,000 genotypes without replacement and then recalculating the statistic. We found that individuals we collected were more genetically related than expected by chance alone. A t-test on the mean relatedness observed and those obtained through a permutation procedure revealed statistically significant differences between them. Observed mean rxy = 0.010 is indicated by an arrow, resampled mean rxy = 0.0071, t = 1173, P<0.001. (TIFF) [file pone.0044953.s001.tiff]

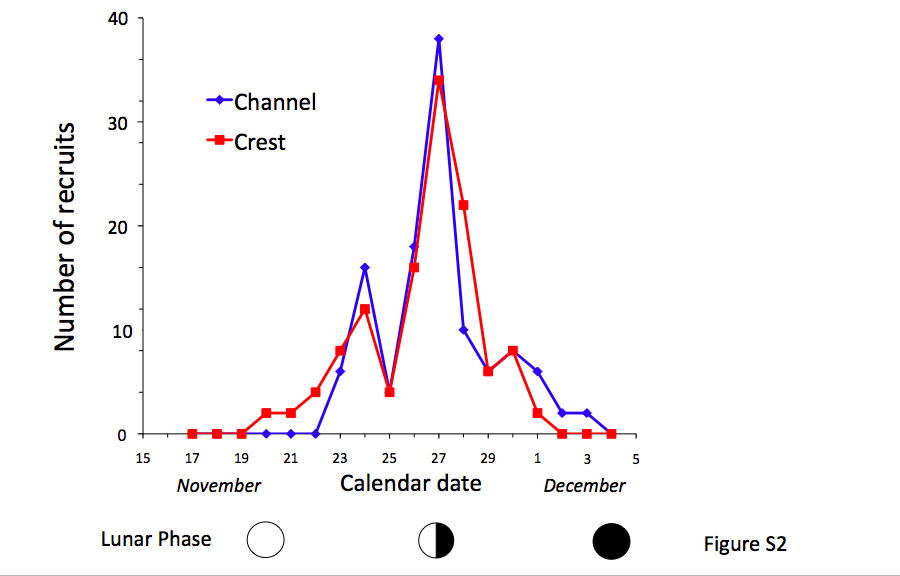

Supplement: Figure S2 — Recruitment of Dascyllus trimaculatus. Plot of number of recruits collected each day of a recruitment cycle in Moorea, French Polynesia. Dates of the month of November and December are on the x axis, number of collected recruits are on the y axis. Phases of the moon are indicated showing a peak recruitment at the quarter moon and a low recruitment at full and new moons. Channel Row and Crest Row recruitments are shown separately in blue and red, respectively. (TIFF) [file pone.0044953.s002.tiff]
